# Composition and Free Radical Scavenging Activity of Kernel Oil from *Torreya g*randis, Carya Cathayensis, and *Myrica Rubra*

**Published:** 2014

**Authors:** Liang Ni, Wei-Yong Shi

**Affiliations:** KeyLaboratory ofPollutedEnvironmentRemediation andEcologicalHealth,MinistryofEducation,CollegeofNaturalResourcesandEnvironmentalScience,ZhejiangUniversity,Hangzhou,China.

**Keywords:** Kernel, Free radical, *Torreya grandis*, *Carya cathayensis*, *Myrica rubra*

## Abstract

In this study, we measured the composition and free radical scavenging activity of several species of nuts, namely, *Torreya grandis*,* Carya cathayensis*, and *Myrica rubra*. The nut kernels of the aforementioned species are rich in fatty acids, particularly in unsaturated fatty acids, and have 51% oil content. *T. grandis *and *C. cathayensis* are mostly produced in ZheJiang province. The trace elements in the kernels of *T. grandis* and* C. cathayensis* were generally higher than those in *M. rubra*, except for Fe with a value of 64.41 mg/Kg. *T. grandis* is rich in selenium (52.91−68.71 mg/Kg). All three kernel oils have a certain free radical scavenging capacity, with the highest value in *M. rubra*. In the DPPH assay, the IC_50_ of *M. rubra* kernel oil was 60 μg/mL, and OH was 100 μg/mL. The results of this study provide basic data for the future development of the edible nut resources in ZheJiang province.

## Introduction

*Torreya grandis* and *Carya cathayensis* are the main edible nuts produced in Zhe Jiang province; these edible nuts have high nutritional value, special texture, and have the ability to cure human diseases, such as ancylostomiasis and filariasis (1). Zhu Ji City produces about 500 tons of *T. grandis* every year, which costs about US$30 per kilogram. It will normally take 2−3 years before becoming edible. *C. cathayensis* is the nut most consumed in Zhe Jiang province, with an annual production of about 13,000 tons in Lin An. With the recent development in the production of *Myrica rubra*, its processing continuously improves in a way similar to that of juice, wine, candied fruit, and so on. A large number of kernel resources of *M. rubra* are produced during processing. According to a survey, there are about 3,000 tons of *M. rubra* kernels wasted in factories every year. If only these wasted resources can be used more efficiently, then the kernels may become a good source of new nuts in Zhe Jiang province. A few of the research studies on the aforementioned nut kernels have been poorly conducted. Thus, the present study on the composition and free radical scavenging activity of the three nut kernels was done to investigate their nutritional condition.

## Experimental

*Chemical* *reagents*

1, 1-diphenyl-2-pycrylhydrazyl (DPPH) was purchased from Sigma Chemical Co. (St. Louis, MO, USA). All the other chemicals and reagents were of the analytical grade and were commercially available.

*Plant materials*

The nut kernels were all collected from ZheJiang province, which is located east of China. Shelled and unsauted *T. grandis* nuts were collected from FuYang (C), ZhuJi (D), and FengQiao (E) in Oct 2010. *M. rubra* nuts were collected form YuYao (F) in Jul 2010, whereas *C. cathayensis *nuts were collected from Fuyang (A) and Linan (B) in Oct 2010. The shells were removed and handcraftedly weighed together with the kernels. Then, the kernels were ground into fine powder.

*Physical and physicochemical characterization*

The physical characterization of the kernel oils was determined in accordance with the method of Ajayi (2). Standard AOAC (3) methods were used to determine the acid, peroxide, and saponification values of the kernel oils. Three different oil samples were analyzed in duplicate.

*Kernel oil extraction*

The method of extracting kernel oils was previously described elsewhere, with some modifications (4). A total of 30 g of kernel powder were combined with 100 mL hexane in a bottle and homogenized for 30 min at room temperature. The mixture was filtered using a filter paper and the residue was re-extracted twice. All the filtrate was mixed and the solvent was removed from the extract using a rotary evaporator at 40 ^o^C. The resulting oil was weighed and transferred into sealed sample vials after it was dried over anhydrous sodium sulphate. The oil was then stored at 4 ^o^C for further assay.

*Gas chromatography-mass spectrometry analysis (GC–MS)*

The GC-MS method for determining the fatty acid content of the nuts was described elsewhere, with some modifications (5-8). A total of 0.25 g kernel oils were dipped in a 2 mL petroleum ether [benzene (V/V=1:1), overnight at room temperature]. A total of 2 mL 0.4 N potassium hydroxide were added in a methanol solution that was homogenized for 1 min. The solution was allowed to stand still for 10 min. The volume was adjusted to 10 mL with saturated sodium chloride for further assay.

Chromatographic separations were performed using a SHIMADZU GC-MS (GC-15A) equipped with a 0.25 μm thick ZB-1 MS fused capillary column (DB-23, 30 m × 0.32 mm *i.d*.). The injector temperature was set at 270 ^o^C. The oven temperature was programmed from 130 to 170^ o^C at 5 ^o^C/min and was held isothermally for 3 min. The temperature was raised to 215 ^o^C at 2.7^ o^C/min, held isothermally for 8 min, and was finally raised to 230 ^o^C at 40^ o^C/min. Helium gas was used as the carrier gas at a constant flow rate of 1 mL/min. A total of 1 μL sample was injected manually in the split-less mode. For MS detection, an electron ionization system with ionization energy of 70 eV was used.

*DPPH radical scavenging activity*

The scavenging effect of the kernel oils was measured in accordance with the method of Gyamfi (9), with slight modifications. A total of 2 mL 0.02 mM (final concentration) DPPH solution in ethanol was briefly mixed with 1 mL of various concentrations of kernel oils (10, 20, 30, 40, 70, 100and150 μg/mL) dissolved in ethanol. Subsequently, the volume was adjusted to 10 mL. The mixture was then vortexed vigorously and left for 60 min at 37 ^o^C in the dark. For the control, a total of 1 mL of ethanol was used, and the absorbance was measured at 517 nm. The scavenging capacity of DPPH is calculated using the following equation:

Scavenging Capacity %=( Ac-As)/Ac*100

where *Ac* is the absorbance of DPPH radical+ethanol, and *As* is the absorbance of DPPH+kernel oil solution.

*Trace elements of kernel powder*

All the plastics and glasswares were cleaned by soaking them with the contact in 10% nitric acid solution overnight, and were then rinsed with deionized water. A 0.5 g sample was digested with 8 mL nitric acid and 1 mL HClO_4_, and then diluted to 100 mL with double deionized water (Milli-Q). The blank digest was carried out in the same way (10). ICP-AES was performed for the determination of trace elements.

*Hydroxyl radical scavenging activity*

The hydroxyl radical scavenging assay was performed in accordance with the method described by Li (11), with slight modifications. Both 1, 10-phenanthroline (1.5 mM) and FeSO_4 _(1.5 mM) were dissolved in phosphate buffer (pH 7.4) and mixed thoroughly. A total of 1 mL H_2_O_2 _(0.01%) and 1 mL of various concentrations of kernel oils (10, 20, 30, 40, 70, and 100 μg/mL) were dissolved in ethanol. Subsequently, the volume was adjusted to 10 mL. The mixture was then vortexed vigorously and left for 60 min at 37 ^o^C in the dark; the absorbance was measured at 536 nm. Hydroxyl radical scavenging activity is calculated using the following equation:

Hydroxyl radical scavenging activity %=( A_s_-A_1_)/ (A_0_-A_1_)*100

where *A*_s_ is the absorbance of the sample; *A*_1_ is the absorbance of control solution containing 2 mL 1, 10-phenanthroling, FeSO_4_, and 1 mL H_2_O_2_; and *A*_0_ is the absorbance of blank solution containing 1, 10-phenanthroline and FeSO_4_.

*Data analysis*

All results are expressed as the means of three separate contents. t-test was used to evaluate significant differences (P < 0.05) among the means for each sample.

## Results and Discussion

*Physical characterization*

The physical characterization of the seeds of *T. grandis, C. cathayensis, *and* M. rubra*, the weight of the seeds and kernels, as well as the oil contents, are shown in Table 1. *C. cathayensis *(A, B) has the largest size among the three nuts and has a weight ranging from 3.42 to 3.81 g/per seed. *M. rubra *(F) has the smallest size with a weight of 0.51 g. The kernel of the nut seed is the main part because it is edible and contains the most nutritious components. As presented in Table 1,* T. grandis *(C, D, and E) has the kernel with the highest percentage. For example, 65.17% of the content of the nut seed of *T. grandis *(B) is kernel. The percentage of kernel from *M. rubra *(F) is the lowest among the three nuts at 11.92%. The oil contents of *C. cathayensis *(A, B) and *M. rubra *(F) are nearly the same, which are 69.94%, 70.37%, and 70.79% for A, B, and C, respectively. Their oil contents are considerably higher than those in *T. grandis *(C, D, E), with percentages of 51.52%, 53.46%, and 55.64%, respectively.

**Table 1:** Physical properties of seeds of *Carya cathayensis, Torreya grandis, *and* Myrica rubra*

	**Weight of seed (g)**	**Kernel of seeds (%)**	**Oil content of kernel (%)**
**A**	3.42±0.14	48.68±0.24	69.94±0.14
**B**	3.81±0.18	46.38±0.21	70.37±0.14
**C**	2.05±0.25	65.17±0.15	51.52±0.52
**D**	1.87±0.25	65.46±0.53	53.46±0.42
**E**	1.67±0.16	63.94±0.54	55.64±0.17
**F**	0.51±0.17	11.92±0.24	70.79±0.16

*Chemical component*

The chemical components of the oil extracts from the three nut seeds are shown in Table 2. The total acidity, which is expressed as acid value, is presented below. *T. grandis *(D) has the highest acid value with 11.35 mg KOH/g oil. *T. grandis *(C, D) is not suitable for edible use because its acid value is higher than 6.0 mg KOH/g oil.* M. rubra *(F) has the lowest acid value of 0.86 mg KOH/g oil, indicating that it has the lowest free acids in oil and may even be a fine source of healthy oils. The peroxide value can express the oxidation level of the oil, and its high peroxide value may be determined using the rancid test. According to a previous study, fresh oils have a peroxide value less than 10 mg/g oil (12). The peroxide values of the three species of nut oils are less than 10 mg/g oil; *T. grandis *(D) has the lowest peroxide value with 1.11 mg/g oil, indicating that *T. grandis *(D) may be stored for a long time without becoming rancid. The saponifcation number can show the molecular weights of the fatty acids. Based on the results, the saponification number ranges from 171.14 to 211.87 mg KOH/g oil. 

**Table 2:** Chemical characterization of seeds of *Carya cathayensis*,* Torreya grandis*, and* Myrica rubra.*

**Species**	**Component**		
	**Acid value** **mg KOH/g oil**	**Peroxide value** **mg/g oil**	**Saponification number** **mg KOH/g oil**
A	2.83±0.13	2.48±0.04	208.51±7.58
B	3.26±0.12	6.18±0.07	190.81±9.53
C	7.29±0.17	5.12±0.13	171.14±2.81
D	11.35±1.17	1.11±0.09	197.07±3.49
E	1.11±0.09	3.23±0.16	188.48±5.19
F	0.86±0.07	5.39±0.37	211.87±5.44

*Fatty acid composition*

The results of kernel oil analysis reveal that the three species of nut oils have substantially high fatty acid contents. The fatty acid composition of these kernel oils are presented in Table 3. All the oil extracts were found to be abundant in unsaturated fatty acids.* T. grandis *(C, D, and E) has the highest value of unsaturated fatty acids, varying from 87.95% to 94.25%. *M. rubra *(F) has an 85.73% unsaturated fatty acid content, whereas *C. cathayensis *(A, B) has 80.14% and 83.55%, respectively. Polyunsaturated fatty acids are very important in daily oil consumption because they reduce the incidence of cardiovascular diseases (13) and cancer (14). In the present study, the value of polyunsaturated fatty acids is similar to that of the unsaturated fatty acids. *T. grandis *(C, D and E) contains the highest percentage of polyunsaturated fatty acids. *M. rubra *(F) has 35.86% polyunsaturated fatty acids in total fatty acids. The value of polyunsaturated fatty acids of* C. cathayensis *(A, B) is almost half of that of *T. grandis *(*C. cathayensis *A, 18.59%) and *M. rubra *(*C. cathayensis *B, 17.70%). In addition, there is evidence that *C. cathayensis *(A, B) is rich in octadecenoic acid, with values of 62.87% and 65.10%, respectively. As mentioned above, the three kinds of nut oils can be a good source of edible oils.

**Table 3:** Fatty acid composition of oil extracts from *Carya cathayensis*,* Torreya grandis*, and* Myrica rubra* (%).

**NO.**	**Fatty acid name**	**Formula**	**Species (%)**					
			**A**	**B**	**C**	**D**	**E**	**F**
1	Tetradecanoate acid	C14H28O2	0.52	0.51	ND	ND	ND	0.11
2	Hexadecenoic acid	C16H30O2	0.43	0.52	ND	ND	ND	0.86
3	Hexadecanoic acid	C16H30O2	15.87	13.9	13.05	17.17	20.73	10.62
4	Heptadecanoic acid	C17H34O2	ND	ND	1.78	6.31	3.48	ND
5	Octadecatrienoic acid	C18H30O2	ND	ND	0.06	0.25	0.20	0.13
6	Octadecadienoic acid	C18H32O2	18.59	17.70	36.29	37.74	35.22	35.07
7	Octadecenoic acid	C18H34O2	62.87	65.10	37.87	31.32	35.56	48.86
8	Octadecanoic acid	C18H36O2	1.46	2.03	3.25	3.06	2.76	3.54
9	Eicosatrienoic acid	C20H34O2	ND	ND	6.31	3.53	1.66	0.19
10	Eicosadienoic acid	C20H36O2	ND	ND	0.88	0.40	0.22	0.47
11	Eicosenoic acid	C20H38O2	0.25	0.24	0.50	0.22	0.16	0.15
	Saturated fatty acid		17.86	16.45	5.75	12.05	8.52	14.27
	Unsaturated fatty acid		82.14	83.55	94.25	87.95	91.48	85.73
	Polyunsaturated fatty acid		18.59	17.70	43.54	41.92	37.30	35.86

ND: not detected

*Trace element of kernel powder*

Trace elements and minerals play a very important role in human health, particularly in biological processes and normal growth and development. Low intake or reduced bioavailability of minerals may lead to deficiencies, which may cause impairment of bodily functions (10). In the present study, we focused on the seven trace elements in kernel powder. The range of each metal in kernel powder is shown in Table 4. *M. rubra *has the poorest Mg and Ca contents, which are 255.08 and 281.36 mg/Kg, respectively. The Mg and Ca in the other kinds of nut are higher compared with those in *Myrica rubra*, in which the values range from 1045.88 to 1994.27 mg/Kg (Mg) and from 948.21 to 1459.74 mg/Kg (Ca). The highest Mg and Ca contents were found in *C. cathayensis *A and *T. grandis *D. From Table 4, the highest microelement Fe has a value of 64.41 mg/Kg in *M rubra*. Based on the results, *T. grandis* is abundant in Co and Se, with values ranging from 0.28 to 0.50 mg/Kg and from 52.91 to 68.71 mg/Kg, respectively. The results show that *C. cathayensis *and *T. grandis *are abundant in Mg and Ca. *T. grandis *is found rich in Co and Se, whereas *Myrica rubra* is high in Fe but low in Mg, Ca, Co, Cu, and Zn.

**Table 4:** Mineral composition of kernel powder from *Carya cathayensis*,* Torreya grandis*, and* Myrica rubra* (mg/Kg).

**Species**	**Element**						
	**Mg**	**Ca**	**Fe**	**Co**	**Cu**	**Zn**	**Se**
A	1994.27	1250.85	7.29	0.10	14.95	50.84	26.08
B	1540.00	1038.41	12.06	0.06	9.09	31.37	19.58
C	1846.82	1030.05	21.01	0.45	14.13	27.33	68.71
D	1045.88	1459.74	22.00	0.28	9.13	65.69	52.91
E	1547.45	948.21	25.00	0.50	15.79	46.45	59.00
F	255.08	281.36	64.41	0.03	1.90	2.20	14.46

Nd: not detected. All values are the means of duplicate determinations

*DPPH scavenging assay*

**Figure 1:**
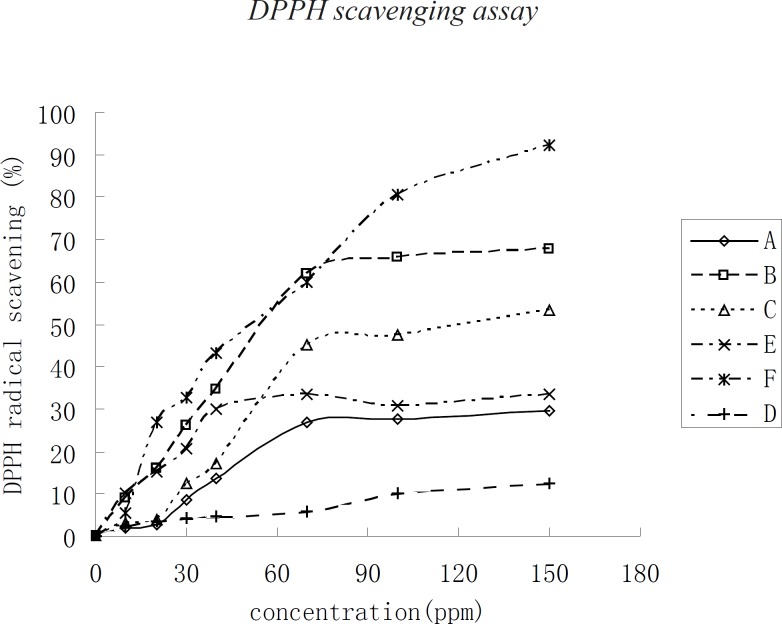
Scavenging of the DPPH radical of oil extracts from *Torreya grandis, Carya cathayensis, *and* Myrica rubra*

DPPH scavenging assay allows the comparison of reactivities of powerful antioxidants such as Vc. The assay of DPPH radical scavenging capacity was used to examine the antioxidant activities of the three kinds of kernel oil extracts. In this assay, F has the greatest DPPH radical scavenging capacity with a kernel oil concentration higher than 70 μg/mL. When the concentration of kernel oil was below 70 μg/mL, the DPPH radical scavenging capacity of the three kinds of kernel oils changed more rapidly with the change in kernel oil concentration. The DPPH radical scavenging capacity then changed slowly when the kernel oil concentration was higher than 70 μg/mL. The DPPH radical scavenging capacity of the kernel oils in decreasing order was F, B, C, E, A, and D, respectively. The concentration of the three kinds of kernel oils required a scavenging capacity of 50% DPPH radical in the medium, which was referred to as IC_50_. Under the assay conditions, the IC_50 _of F, D, and B were 60 μg/mL, followed by C with an IC_50_ of 120 μg/mL. The IC_50_ of E, A, and D were higher than 120 μg/mL. The DPPH radical scavenging capacity of the three kinds of kernel oil has not been investigated yet. The antioxidant activity of the three kinds of kernel oil is due to their chemical compositions, similar to those of phospholipids, α–tocopherol, and so on.

**Figure 2:**
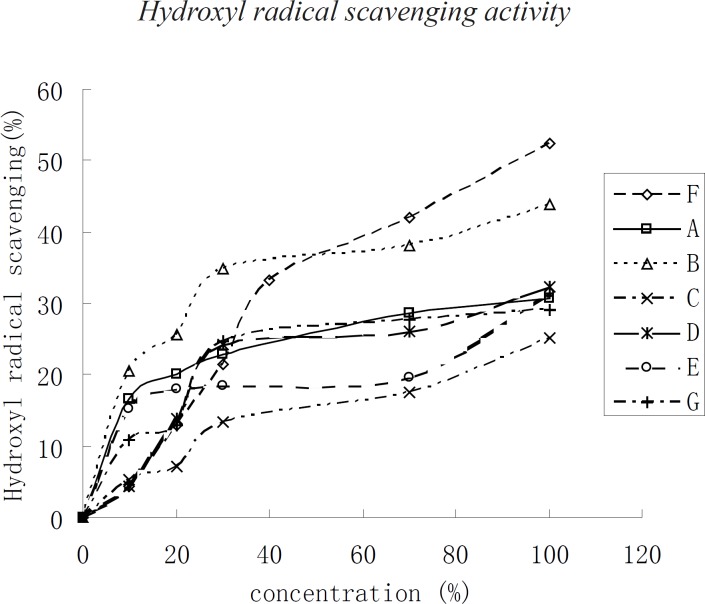
Scavenging of the hydroxyl radical of oil extracts from *Torreya grandis*,* Carya cathayensis*, and* Myrica rubra*

*Hydroxyl radical scavenging activity*

The trend of hydroxyl radical scavenging was nearly the same as that of DPPH when the concentration of kernel oil was below 40 μg/mL. The hydroxyl radical scavenging capacity of the three kinds of kernel oils changed more rapidly. In contrast, when the concentration was higher than 40 μg/mL, the scavenging capacity changed slowly according to the concentration of the kernel oil. In the present assay, the IC_50 _of F was 100 μg/mL, followed by the other kernel oil with an assay higher than 100 μg/mL.

## Conclusions

Based on the results of the present assay and according to literature, the three kinds of nut kernels of interest are very nutritional. These kinds of nut kernels have substantially high fatty acid contents. The highest oil content of kernel was found in F with a value of 70.79%, which is similar to that in previous studies. All the three kernel oils have a high content of unsaturated fatty acids. *T. grandis *and* M. rubra *are especially abundant in polyunsaturated fatty acids, indicating that their oils are healthful and thus can be used for cooking. The trace elements are different. *M. rubra *has a low content of trace elements, except Fe. *T. grandis *has a high rate of Se. These kinds of kernel oils all have radical scavenging capacities. The highest DPPH radical scavenging capacity was found in F, indicating that *M. rubra *has better radical scavenging capacity. In summary, the three kinds of nut kernels grown in Zhejiang province are healthful and nutritious.
